# Perception of usefulness of laboratory tests ordering by internal medicine residents in ambulatory setting: A single-center prospective cohort study

**DOI:** 10.1371/journal.pone.0250769

**Published:** 2021-05-11

**Authors:** Dimitria Doi, Romulo Ribeiro do Vale, Jean Michell Correia Monteiro, Glauco Cabral Marinho Plens, Mario Ferreira Junior, Luiz Augusto Marcondes Fonseca, Sandro Félix Perazzio, Bruno Adler Maccagnan Pinheiro Besen, Arnaldo Lichtenstein, Leandro Utino Taniguchi, Nairo Massakazu Sumita, Aline Pivetta Corá, Adriana Pasmanik Eisencraft, Alberto José da Silva Duarte

**Affiliations:** 1 Central Laboratory Division, Hospital das Clínicas HCFMUSP, Faculdade de Medicina, Universidade de São Paulo, São Paulo, SP, Brazil; 2 Department of Internal Medicine, Hospital das Clínicas HCFMUSP, Faculdade de Medicina, Universidade de São Paulo, São Paulo, SP, Brazil; 3 Division of Rheumatology, Universidade Federal de São Paulo, São Paulo, SP, Brazil; 4 Medical ICU, Disciplina de Emergências Clínicas, Departamento de Clínica Médica, Hospital das Clínicas HCFMUSP, Faculdade de Medicina, Universidade de São Paulo, São Paulo, SP, Brazil; 5 Emergency Department, Children’s Institute, Hospital das Clínicas HCFMUSP, Faculdade de Medicina, Universidade de São Paulo, São Paulo, SP, Brazil; Universidade de Mogi das Cruzes, BRAZIL

## Abstract

The demand for high value health care uncovered a steady trend in laboratory tests ordering and inappropriate testing practices. Residents’ training in laboratory ordering practice provides an opportunity for quality improvement. We collected information on demographics, the main reason for the appointment, preexisting medical conditions and presence of co-morbidities from first-visit patients to the internal medicine outpatient service of our university general hospital. We also collected information on all laboratory tests ordered by the attending medical residents. At a follow-up visit, we recorded residents’ subjective perception on the usefulness of each ordered laboratory test for the purposes of diagnosis, prognosis, treatment or screening. We observed that 17.3% of all ordered tests had no perceived utility by the attending resident. Tests were usually ordered to exclude differential diagnoses (26.7%) and to help prognosis estimation (19.1%). Age and co-morbidity influenced the chosen category to legitimate usefulness of tests ordering. This study suggests that clinical objectives (diagnosis, prognosis, treatment or prevention) as well as personalization to age and previous health conditions should be considered before test ordering to allow a more appropriate laboratory tests ordering, but further studies are necessary to examine this framework beyond this medical training scenario.

## Introduction

According to the Lewin report, laboratory medicine is an “essential element of the health care system, integrated to many clinical decisions, providing physicians, nurses, and other health care providers with often pivotal information for prevention, diagnosis, treatment, and management of disease” [1]. As such, laboratory tests are frequently ordered during medical evaluation in different settings and an increase in ordering has been observed (for example, an annual increase of 8.7% has been described in the United Kingdom primary care) [2]. Although they are useful in clinical practice, inappropriate testing and demands for high value care have aroused considerable attention in recent years [3–5].

Laboratory tests are important tools in health care, playing an important auxiliary role in disease diagnosis and prognosis, as well as in risk stratification, treatment choice and assessment of the response to it, and monitoring of the course of disease [6]. Bindraban et al estimate a 20% excessive laboratory tests order [7], but the prevalence of inappropriate testing remains unknown. Inappropriate herein means not only overutilization by ordering unneeded tests, but also underutilization by not ordering needed tests. Other instances of inappropriate testing can occur during the initial evaluation of the patient or in response to new signs and symptoms; the routine unadvised repetition of tests for the same patient is still another example of inappropriate testing. Despite the background of inappropriate testing, this approach is potentially harmful and may induce medical errors, unnecessary lengthening of hospital admissions and even imply legal issues. Additionally, overutilization may induce other unnecessary auxiliary diagnostic procedures and increase false-positive results, erroneous diagnoses, costs rising, adverse events and number of phlebotomies, which, in turn, culminate with anemia, mainly in patients under intensive care. On the other hand, underutilization may lead to a worse clinical outcome due to diagnostic delay [8].

In the hospital daily routine, a common “panel” of tests is ordered daily for admitted patients (e.g. complete blood count, electrolytes, renal function), often regardless of patients’ clinical status and of previously normal results. Even if one can argue that the waste incurred by routinely ordered tests is small, when compared with the total health care costs, financial and clinical costs become significant when they add up to each other [6]. According to the literature, medical residents are more prone than senior doctors to order tests inappropriately, a finding attributed to the routines of services, lack of knowledge of costs, diagnostic uncertainty and to the very expectancy of daily tests orders by the residents’ mentors, as previously described by Sedrak et al [9]. Hence, contributing factors for the overutilization of laboratory tests include the practice of defensive medicine, the expectations of patients, insufficient understanding of the limitations of tests, inability to retrieve the results of previous tests, deeply rooted behaviors and financial incentives [10].

The adequacy of a laboratory test is often based on experts’ opinion and on evidence about the performance characteristics of the test (for example, its sensitivity and specificity). Including the outcome of patients submitted to the test can broaden the scope of that discussion. For example, analyzing which further tests, invasive procedures or treatments were requested or aborted based on the test results and evaluating its impact on the clinical outcome, quality of life and on survival of patients. Clues to answer such questions are few or non-existent in the literature [11].

Australian studies from 2006 showed that 67.9% (2.01 tests per patient-day) of the laboratory tests performed during a 6-months period did not contribute to patient care [10]. The efficacy of the interventions destined to support the proper use of tests is not clear, as well as the gaps in the current state of research on the subject, despite its presence on the literature [12]. In the 1980’s Grossman reviewed five intervention strategies destined to curb the high burden of laboratory tests: educational strategies, comparative feedback between medical orders and the protocols available, cost consciousness, rationing of resources and financial incentives [13]. More recently, international campaigns aiming at the unnecessary use of tests and procedures have been launched. The United Kingdom launched the recommendations of the campaign *Do Not Do*; in the United States, the *Choosing Wisely* campaign was conceptualized with the mission to provide guidance on more appropriate medical tests ordering, treatments and procedures [7].

Notably, appropriate laboratory testing education is lacking in many medical schools [14, 15]. This is particularly worrisome, since interns and residents are a relevant source of variation in ordering practice [16], and university-affiliated institutions should promote high-value care. In our internal medicine ward (located in a university-affiliated public tertiary hospital in Brazil), we observed that repetition of tests was a common practice, regardless of patients’ diagnosis or previous results [4]. Although residents frequently acknowledge that unnecessary routine laboratory tests ordering is common practice, they also claim that they did not receive instruction in such practice [9]. Therefore, residents’ training of laboratory ordering practice provides a clear opportunity for improvements in medical curriculum.

The main objectives of this study are: (1) to describe residents’ perception of the role of each laboratory test during outpatient management, (2) to associate this role with patients’ characteristics.

## Materials and methods

### Study settings and participants

We conducted a prospective single-center study in the Internal Medicine outpatient clinic from Hospital das Clínicas of University of São Paulo Medical School (HCFMUSP), a public tertiary-center in São Paulo, Brazil. Further description of our hospital is available in a previous publication [4]. Briefly, our academic outpatient clinic is focused on fast-track appointments of either undiagnosed patients or those carrying yet uncontrolled diseases, referred from different intramural or extramural services. The study was conducted from August to December, 2018. All first year Internal Medicine residents were invited to participate in this test ordering quality improvement program during their outpatient services rotation. We collected data during medical appointments of non-oncological patients referred to our service. We considered first office visit patients seen during the study period as a single inclusion criterion. No exclusion criteria were considered. The institutional review board (IRB), Human Subject Research, Research Ethics Committee of HCFMUSP approved the study (number 2.160.459) and waived the need for informed consent, since this initiative is part of a quality improvement program of the Central Laboratory Division of HCFMUSP.

### Data collection

A study author collected the data generated in each patient office visit, including patients’ age and gender, referring service, main objective of the first visit (either “Diagnostic investigation”, for undiagnosed patients; or “Disease control”, for those carrying yet uncontrolled, though previously diagnosed, chronic diseases), preexisting medical conditions and co-morbidities (i.e. Charlson co-morbidity index) [17], and destination on discharge. We also collected detailed information on the laboratory tests ordered during office visit. After the follow-up visit, we registered the subjective perception of the attending resident on the usefulness of each laboratory test for the purposes of: (1) “Diagnosis”, if a given test or panel of exams helped either confirm a specific diagnosis hypothesized in the first visit, or order other tests or exclude a differential diagnosis; (2) “Prognosis”, if a given test or panel of exams unmistakably allowed risk factor calculation of either specific diseases or targeted organ damage; (3) “Treatment”, defined by the presence of a test or a panel of tests that influenced patient therapy and/or management, including advice on lifestyle changes; or (4) “Prevention”, if a given test or panel of exams was ordered to track possible age or gender-related silent diseases, or was unmistakably responsible to establish a specific diagnosis which was not formulated in the first visit, or even indicated prophylactic medications or vaccine administration. A single test could fit in more than one classification. If the test had not yielded any information for any chosen category to legitimate its usefulness, we labeled it as ‘none’. Every ordered test was registered on a form (S1 Appendix), which was completely fulfilled during the next visit by the medical resident himself, using patients self-informed real-time information and respective medical records simultaneously. Residents order lab tests based on their own clinical judgment and their perceptions were exclusively assessed at the return of results during follow-up visit.

Before data collection, we tested preliminary versions of the collecting instrument with voluntary medical residents, in order to clarify doubts, inconsistencies and flaws. In addition. every modification of those preliminary versions was weekly discussed among all coauthors to validate the final version of the form applied in the study, which, in turn, is available online as (S1 Appendix). Data was collected on paper and then electronically stored on the RedCap® platform.

### Data analysis and sample size calculation

We did not perform a priori sample size calculation. However, assuming a 50% proportion of tests with no utility (the most conservative estimate for a proportion), three hundred and eighty four tests at follow-up would be necessary to provide a 5% margin of error in proportion estimation, with a 95% confidence interval. With a 3% margin of error, 1,067 tests would be necessary with the same assumptions. We estimated an average of 10 tests per patient in follow-up. Therefore, 39 patient encounters would be necessary with the less precise margin of error and 107 would be necessary for the 3% margin of error. Given the outpatient clinic number of consultations, we decided to include patients in a five-month period, which would be enough to provide estimates for the number of tests with no utility with the desired precision.

Descriptive data are presented as mean ± standard deviation or median and inter-quartile range, according to data distribution. Frequencies are presented as the number (and percentage) of tests by each item or group of items classified according to purpose of test ordering, patient age and tertiles of Charlson index. We did not perform classical hypothesis testing.

## Results

**Fig 1:**
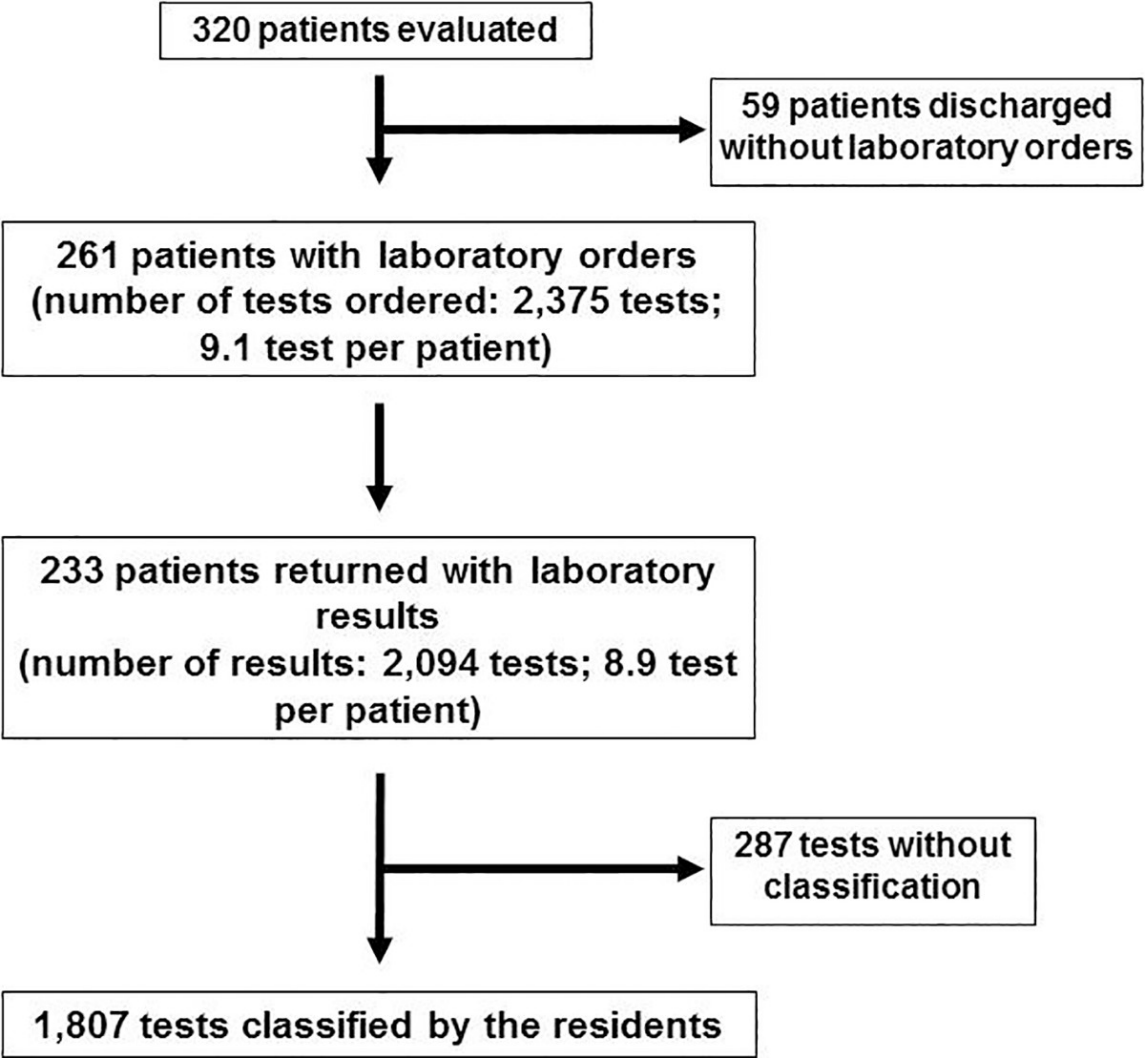
Flowchart of patient evaluation and enrolment.

Thirty-six 1^st^ year residents (out of 72 eligible during the study period) participated in this study during their outpatient clinic rotation. Twenty were male (55%) and their mean age was 25.5 years, ranging from 22 to 31 years-old. We included 320 patients in the study, sequentially referred to our service; mean age was 55.5 ± 17.7 years, 43.8% were male and median Charlson index was 2 (0–4). Most of the office visits were for investigation purposes (195/320, 60.9%), and 125 were for disease control (125/320, 39.1%) (Table 1). Fifty-nine of the 320 patients were discharged without laboratory test orders (18.6%). On the follow-up visit, 233 patients had performed 2,094 laboratory tests (8.9 tests per patient); however, 287 tests were not classified by the attending resident, leaving 1,807 laboratory tests to be evaluated (Fig 1).

**Table 1:** Baseline patient characteristics.

Characteristic	N = 320
Age, mean (SD)	55.5 (17.7)
Male sex, n (%)	140 (43.8%)
Charlson comorbidity score, median [P25, P75]	2 [0, 4]
1^st^ appointment number of exams, median [P25, P75]	6 [1, 12]
Patient referral, n (%)	
*Emergency Department*	256 (80%)
*Same hospital–other specialty*	37 (11.6%)
*Primary care*	3 (0.9%)
*Other*	24 (7.5%)
Purpose of 1^st^ appointment, n (%)	
*Diagnostic investigation*	195 (60.9%)
*Disease control*	125 (39.1%)
Patient destination, n (%)	
*Discharged*	34 (10.6%)
*Emergency department*	4 (1.3%)
*Ward admission*	11 (3.4%)
*Follow-up at same outpatient service*	151 (47.2%)
*Referral to another specialty outpatient service*	63 (19.7%)
*Primary care*	13 (4%)
*Other*	44 (13.8%)

**Fig 2:**
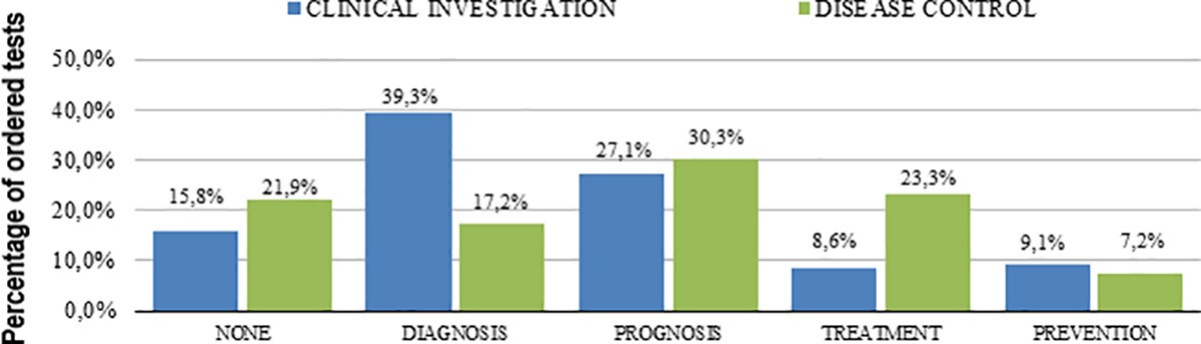
Tests usefulness according to reason for office visit.

**Fig 3:**
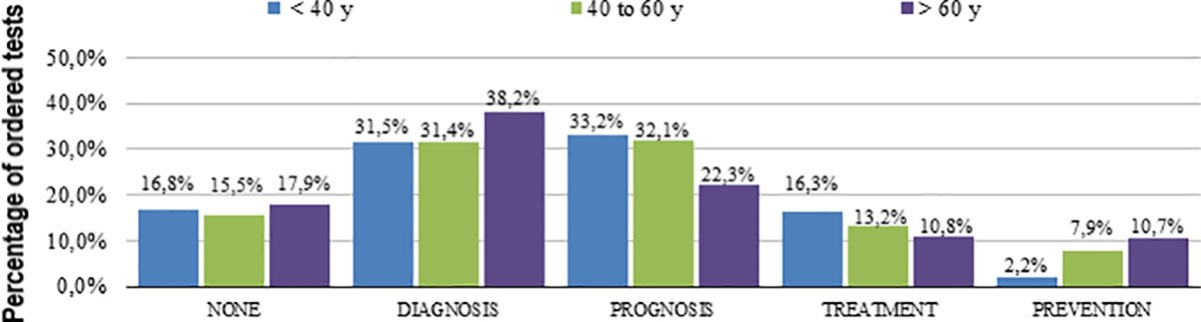
Tests usefulness according to patients’ age. CCI: Charlson index.

**Fig 4:**
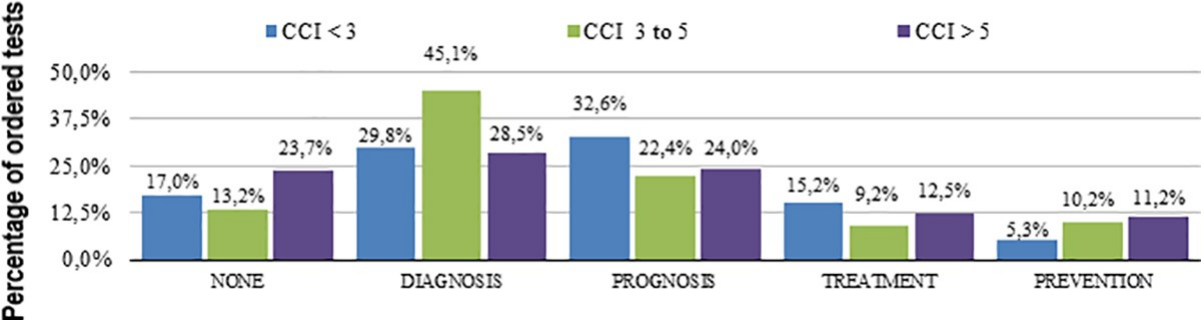
Tests usefulness according to bands of the Charlson Comorbidity Index (CCI).

We found that 313 tests had no perceived utility by the attending resident (17.3% of all evaluated tests). From Table 2 (which presents the perceived utility of tests), the most common uses were to exclude differential diagnoses and to help prognosis estimation (Fig 2). Table 2 clearly shows the difference on test ordering utility, especially whether the main purpose relied on diagnosis or disease control. Therefore, the exclusion of a diagnosis was more frequently observed in Diagnostic investigation (DI) group, whereas targeted organ damage evaluation was more common in Disease control (DC) group. Age and co-morbidity influenced the classification of the tests (Figs 3 and 4).

**Table 2:** Tests ordered and categorized after follow-up, according to the main objective of initial visit.

Test utility	MAIN OBJECTIVE OF INITIAL VISIT					
	Diagnostic investigation (DI)		Disease control (DC)		TOTAL	
	N	(%)	N	(%)	N	(%)
**NONE**	216	(15.8)	97	(22.0)	313	(17.3)
**DIAGNOSIS**						
Conclude diagnostic investigation	90	(6.6)	5	(1.1)	95	(5.3)
Order another test	23	(1.7	13	(2.9)	36	(2.0
Exclude differential diagnoses	424	(31.1)	58	(13.1)	482	(26.7)
**PROGNOSIS**						
Prognostic estimate	276	(20.2)	69	(15.6)	345	(19.1)
Evaluate involvement of target-organ	94	(6.9)	65	(14.7)	159	(8.8)
**TREATMENT**						
Keep current treatment	51	(3.7)	59	(13.4)	110	(6.1)
Change current treatment	37	(2.7)	28	(6.3)	65	(3.6)
Withdraw current treatment	1	(0.1)	4	(0.9)	5	(0.3)
Start new treatment	24	(1.8)	6	(1.4)	30	(1.7)
Advise healthy habits	5	(0.4)	6	(1.4)	11	(0.6)
**PREVENTION**						
Screening of disease that will be treated	116	(8.5)	26	(5.9)	142	(7.9)
Screening of disease that will not be treated	6	(0.4)	6	(1.4)	12	(0.7)
Prescribe prophylactic medicine or vaccine	2	(0.2)	0	(0.0)	2	(0.1)
**TOTAL**	1365	(100)	442	(100)	1807	(100)

We evaluated separately the most commonly ordered tests (responsible for 80% of all tests evaluated). They varied according to the perceived clinical contribution. Table 3 discriminates each test utility and shows the less useful tests: serum sodium, C reactive protein, serum phosphorus and free T4. Lipid profile was the most frequently test ordered for “Prevention”, whereas complete blood count was usually ordered for differential diagnosis exclusion (“Diagnosis”). Interestingly, some specific tests were simultaneously frequent within more than one category: glycated hemoglobin was commonly ordered for disease control (“Treatment”) and for long-term risk factor calculation (“Prognostic”); HIV and B and C hepatitis serologies were used not only for diagnostic purposes but also to prevent diseases.

**Table 3:** Clinical contribution of the tests most frequently ordered and ranked by physicians.

Laboratory Test	Clinical Contribution											
	None		Diagnostic		Prognostic		Treatment		Prevention		Total	
	N	(%)	N	(%)	N	(%)	N	(%)	N	(%)	N	(%)
1. Complete blood count	30	(20.4)	58	(39.5)	43	(29.3)	11	(7.5)	5	(3.4)	147	(100)
2. Tests of renal function (creatinine, BUN)	19	(17)	16	(14.3)	63	(56.3)	10	(8.9)	4	(3.6)	112	(100)
3. Potassium	25	(24)	13	(12.5)	39	(37.5)	26	(25)	1	(1)	104	(100)
4. Sodium	37	(42)	6	(6.8)	29	(33)	16	(18.2)	0	(0)	88	(100)
5. Lipid profile	6	(8.1)	5	(6.8)	16	(21.6)	22	(29.7)	25	(33.8)	74	(100)
6. TSH	5	(7.1)	41	(58.6)	4	(5.7)	11	(15.7)	9	(12.9)	70	(100)
7. Urinalysis	10	(16.7)	22	(36.7)	26	(43.3)	2	(3.3)	0	(0)	60	(100)
8. INR	5	(8.6)	18	(31)	10	(17.2)	25	(43.1)	0	(0)	58	(100)
9 Glycated hemoglobin	6	(11.1)	8	(14.8)	13	(24.1)	19	(35.2)	8	(14.8)	54	(100)
10. HIV (antibodies)	0	(0)	33	(62.3)	1	(1.9)	0	(0)	19	(35.8)	53	(100)
11. Syphilis (antibodies)	1	(1.9)	31	(59.6)	1	(1.9)	2	(3.8)	17	(32.7)	52	(100)
12. Total calcium	12	(24)	17	(34)	15	(30)	4	(8)	2	(4)	50	(100)
13. AST and ALT	12	(24)	15	(30)	18	(36)	5	(10)	0	(0)	50	(100)
14. Fasting glucose	8	(16.7)	10	(20.8)	4	(8.3)	11	(22.9)	15	(31.3)	48	(100)
15. HBV (antibodies)	2	(4.3)	27	(57.4)	1	(2.1)	1	(2.1)	16	(34)	47	(100)
16. HCV (antibodies)	1	(2.2)	27	(60)	1	(2.2)	0	(0)	16	(35.6)	45	(100)
17. CRP	17	(37.8)	13	(28.9)	12	(26.7)	3	(6.7)	0	(0)	45	(100)
18. Free T4	15	(35.7)	13	(31)	3	(7.1)	9	(21.4)	2	(4.8)	42	(100)
19. GGT and alkaline phosphatase	5	(13.9)	13	(36.1)	13	(36.1)	4	(11.1)	1	(2.8)	36	(100)
20. Ferritin	2	(5.6)	21	(58.3)	7	(19.4)	5	(13.9)	1	(2.8)	36	(100)
21. Albumin	6	(17.1)	10	(28.6)	19	(54.3)	0	(0)	0	(0)	35	(100)
22. Serum iron	5	(15.2)	20	(60.6)	4	(12.1)	4	(12.1)	0	(0)	33	(100)
23. Total iron binding capacity	4	(13.8)	19	(65.5)	2	(6.9)	4	(13.8)	0	(0)	29	(100)
24. Bilirrubin	1	(3.6)	11	(39.3)	14	(50)	2	(7.1)	0	(0)	28	(100)
25. Phosphorus	9	(32.1)	6	(21.4)	11	(39.3)	1	(3.6)	1	(3.6)	28	(100)
26. Uric acid	2	(7.4)	9	(33.3)	12	(44.4)	3	(11.1)	1	(3.7)	27	(100)

BUN: blood urea nitrogen; TSH: thyroid stimulating hormone; INR: international normalized ratio; HIV/HBV/HCV: serologic diagnosis for HIV, HBV and HCV; AST: aspartate aminotransferase; ALT: alanine aminotransferase; CRP: C-reactive protein; GGT: Gama-glutamil transferase

## Discussion

In the current study, the mean number of tests per patient was 9.1, which is not high for a first office visit. A previous study from our group reported a mean of 9.5 tests per day for hospitalized patients [4]. Continuing medical education should be the path for behavioral change towards a more appropriate use of tests, since there still is considerable waste in tests orders: in our study, residents deemed 17.3% of them useless. The laboratory tests most frequently deemed of no utility were serum sodium (42%), C-reactive protein (37.8%), free T4 (35.7%) and serum phosphorus (32.1%), followed by aminotransferases (24%) and complete blood count (20.4%). The perceived utility was not different according to the patient’s age neither Charlson comorbidity index. These results may help quality improvement initiatives focusing on a more rational use of laboratory tests during medical training.

This study reveals the perceived usefulness of each test, according to patients’ profile. This seems reasonable, but on a previous paper, we showed that the same tests were systematically ordered, regardless of the patient’s diagnosis [4]. Diagnostic hypothesis-driven, therapeutic decision-based or primary prevention test ordering strategies should be a norm for lab test requests, that unfortunately is not followed by most physicians, especially those in training.

Different laboratory tests may have different perceived utilities, as we observed in our results. For example, a complete blood count can help indicating and differentiating among a number of hematological phenotypes, such as iron deficiency, megaloblastic anemia, beta-thalassemia, leukemias, febrile neutropenia, etc. Similarly, abnormal creatinine clearance diagnoses chronic kidney disease and helps establish its stage [18], which, in turn, ultimately can define a patient prognosis. Other examples of more specific usefulness of tests are the serologic tests for the diagnoses of syphilis and HIV infection (Table 3). Conversely, our group previously demonstrated that C reactive protein has limited utility in an outpatient setting [19]. Electrolytes, like sodium and phosphorus, proved useless in about one third of the time. It is noteworthy that an inertial behavior, consisting in ordering potassium always paired with sodium, and also ordering an electrolytes profile, including calcium, phosphorus and magnesium, without a real need, persists. An auspicious finding was the fall in ionized calcium ordering, a situation that was completely different a few years ago when that electrolyte ranked among the 80% more frequently ordered tests [4].

Designing a specific test-based algorithm is one possible approach for better lab test ordering, as previously suggested by our group [19], but this still needs further validation. An alternative is a clinical diagnosis-driven algorithm development. However, our cohort was made up of by patients with multiple comorbidities, as indicated by a high Charlson index, which could make these approaches hard to implement by obligating the adoption of several algorithms simultaneously. In this scenario, checklists before each test order can help physicians reflet on the real neeed of each test. Our study attempted to address this issue by creating a clinical categorization: diagnostic, prognostic, treatment and prevention. The relative contributions of each of these categories were different according to the patient’s burden of comorbidities, age and sex. Therefeore, this framework could be helpful in medical teaching to increase physician-in-training awareness of the potential utility of their ordered laboratory tests, a recommended domain of the competences of systems-based practice and practice-based learning and improvement. This could lead to more sensitive test ordering and limit unnecessary tests.

Our study has some limitations. First and mainly, it was not blinded and, hence, the assisting resident may have been biased toward a careful self-judgment test ordering. Although this could have biased the estimate downward, 17.6% of all tests were still perceived as “useless” and represent an opportunity for improvement of medical residents’ behavior in test ordering, especially among the tests most frequently considered of no utility. Another limitation is that we could not adjust our analyses for clustering. We also neither intended to deepen into cost-efficacy discussion nor to address economic impact on test ordering. Additional studies are necessary to evaluate necessary unordered tests, which may also impact on final health costs. This study also has not compared different strategies to optimize rational lab test ordering. Our objective was to initially shed some light onto this subject. Indeed, we believe that the categorization herein presented may help on determining lab test real clinical utility and, ultimately, also guide ordering decision. Finally, our results may not be generalizable to all outpatient settings, since it was conducted in a tertiary academic medical center with residents in training.

## Conclusion

Medical residents perceived that roughly 20% of their ordered tests were of no clinical utility, with some variability according to the ordered test. We suggest that a framework considering the intended clinical objectives (diagnosis, prognosis, treatment or prevention) are important questions that should be routinely considered before thoughtless test ordering. This strategy needs further studies to assess its utility beyond the scenario of medical education.

## Supporting information

S1 Appendix Final version of the form applied in the study to categorize lab tests ordered. (TIF)
