# Supplementary figures and images for: Perception of usefulness of laboratory tests ordering by internal medicine residents in ambulatory setting: A single-center prospective cohort study
Source: PLoS One. 2021 May 11;16(5):e0250769. doi: 10.1371/journal.pone.0250769 (PMC8112663; doi:10.1371/journal.pone.0250769)

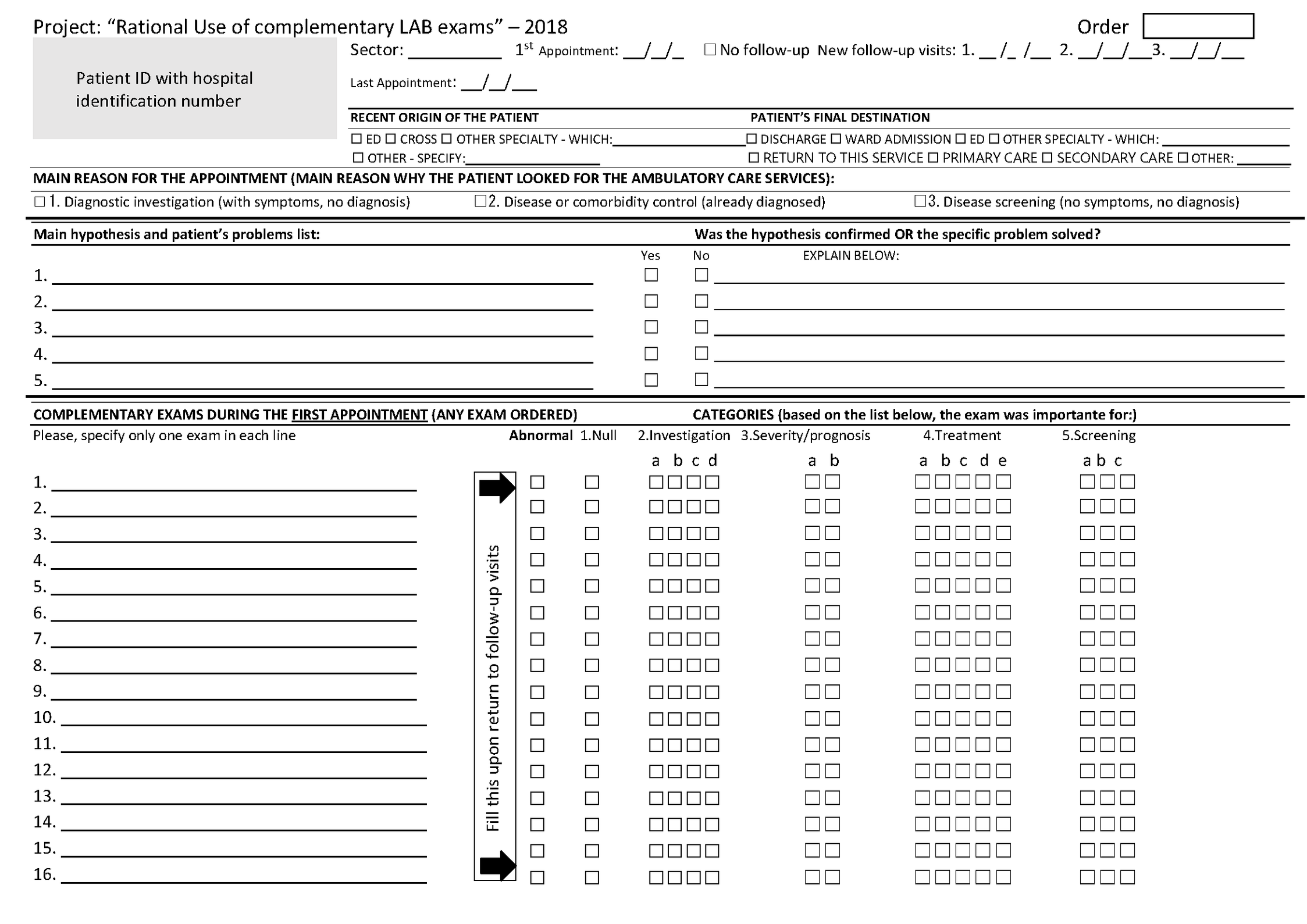

Supplement: S1 Appendix — (TIF) [file pone.0250769.s001.tif]
